# Isolation and Identification of Plant Growth Promoting Rhizobacteria from Sago Palm (*Metroxylon sagu*, Rottb.)

**DOI:** 10.21315/tlsr2021.32.3.3

**Published:** 2021-09-30

**Authors:** Samuel Lihan, Flonia Benet, Awang Ahmad Sallehin Awang Husaini, Kasing Apun, Hairul Azman Roslan, Habsah Hassan

**Affiliations:** 1 Institute of Biodiversity and Environmental Conservation, Universiti Malaysia Sarawak, 94300 Kota Samarahan, Sarawak, Malaysia; 2 Faculty of Resource Science and Technology, Universiti Malaysia Sarawak, 94300 Kota Samarahan, Sarawak, Malaysia

**Keywords:** Plant Growth Promoting Rhizobacteria, *Metroxylon sagu*, Rottb, IAA, Phosphate Solubilisation, Biofertilisers, Rizobakteria Penggalak Pertumbuhan Tumbuhan, *Metroxylon sagu*, Rottb, IAA, Pengurai Fosfat, Biobaja

## Abstract

Plant growth promoting rhizobacteria (PGPR) are strains of naturally occurring soil bacteria that live in close vicinity to the plant’s rhizosphere region which possess the capability to augment host growth. This study was conducted to isolate and identify potential PGPR isolates indigenous to *Metroxylon sagu*, Rottb. rhizosphere. These potential isolates were characterised based on their beneficial plant growth promoting (PGP) properties and identified by molecular analysis via 16S rDNA sequencing. A total of 18 isolates were successfully isolated, out of which five isolates were tested, and designated as (S1A, S2B, S3A, S3C and S42). Among the five isolates, two isolates (S2B and S3C) were found to produce high levels of indole-3-acetic acid (2.96 μg/mL and 10.31 μg/mL), able to fix nitrogen and show significant activity in phosphate solubilisation. The analysis of their sequences via National Center for Biotechnology Information (NCBI) suggested their close identity towards *Lysinibacillus sphaericus* and *Bacillus thuringiensis*. It can be concluded that the isolated PGPR possesses beneficial PGP attributes. It can be implied that the isolated PGPR are potential to be used as inoculant biofertilisers, beneficial for *Metroxylon sagu*, Rottb. growth. Hence, further studies need to be done to evaluate the effectiveness of the beneficial microbes towards sago seedlings growth, under pot experiment.

## Abstract (fr)

Rizobakteria penggalak pertumbuhan tumbuhan adalah sejenis bakteria tanah yang hidup berdekatan rizosfera tumbuhan dan mempunyai impak berfaedah ke atas pertumbuhan tanaman. Kajian ini telah dijalankan untuk melakukan pengasingan dan pengecaman PGPR, daripada rizosfera *Metroxylon sagu*, Rottb. Bakteria endofit diasing berdasarkan ciri-ciri penggalak tumbuhan yang kemudiannya dipilih untuk pengenalpastian secara biologi molekul melalui *16S rDNA sequencing*. Sebanyak 18 bakteria berjaya diasingkan, dan lima jenis bakteria dipilih berdasarkan ciri-ciri penggalak tumbuhan dan dilabelkan sebagai S1A, S2B, S3A, S3C dan S42. Di antara kelima-lima bakteria tersebut, dua bakteria (S2B dan S3C) dijumpai mempunyai kemampuan untuk menghasilkan kadar *indole-3-acetic acid* (IAA) yang tinggi (2.96 μg/mL dan 10.31 μg/mL), mempunyai kemampuan mengikat gas nitrogen serta menunjukkan aktiviti yang memberangsangkan dalam mengurai fosfat. Hasil analisis biologi molekul melalui NCBI menunjukkan identiti terdekat kedua-dua bakteria tersebut cenderung kepada *Lysinibacillus sphaericus* dan *Bacillus thuringiensis*. Melalui hasil penyelidikan ini, dapat dirumuskan bahawa PGPR yang telah diasingkan daripada rizosfera *Metroxylon sagu*, Rottb., mempunyai ciri-ciri penggalak tumbuhan dan berpotensi untuk digunakan sebagai inokulan biobaja, berfaedah kepada pertumbuhan *Metroxylon sagu*, Rottb. Maka, kajian selanjutnya perlu dilakukan untuk menilai keberkesanan mikrob ke atas pertumbuhan benih sagu, melalui eskperimen dalam pasu.

**Highlights**

- Five PGPR isolated from *Metroxylon sagu*, Rottb. rhizosphere were tested on their plant growth promoting traits.
- Among the isolates, *Lysinibacillus sphaericus* and *Bacillus thuringiensis* were found to show significant activity in nitrogen fixing, producing IAA and solubilising phosphate.
- The study implied the isolated PGPR potentially to be used as inoculant biofertilisers, beneficial for *Metroxylon sagu*, Rottb. growth.

## INTRODUCTION

Sago palm (*Metroxylon sagu*, Rottb.) is one of typical underutilised indigenous food crops found in Asia and Pacific Region (Konuma *et al*. 2012). *M. sagu* is most productive plant in comparison with other carbohydrate-producing crops (Bintoro *et al*. 2018). Sago industry in Malaysia, specifically in Sarawak is well established and becoming one of reliable industry contributing to export revenue. Ming *et al*. (2018) stated that Malaysia is the third main sago producer in the world, after Indonesia and Papua New Guinea. In Malaysia, the sago-planting area concentrated in Sarawak, covered approximately 19,702 ha, cultivated in wild and semi-wild conditions (Amin *et al*. 2019). Despite of not being the world’s sago producer, Sarawak reported to be one of the world’s sole sago exporter. Annually, the state has exported more than 40,000 tons to different countries, including to Peninsular Malaysia, Singapore, Taiwan and Japan (Amin *et al*. 2019). This number is expected to rise as there is an increment in starch production as well as in export value, at 15% to 20% every year (Amin *et al*. 2019).

Despite all the positive attributes brought by *M. sagu*, there is rising concerns, whereby upon high biomass production of *M. sagu*, the soil nutrients might deplete over the years. Thus, in order to promote productivity, an emergence in wide application of chemical inputs such as fertilisers, herbicides, fungicides and insecticides, was observed in agriculture (Gou *et al*. 2020). Nevertheless, extended use of these products has often brought adverse effects on the complex system of biogeochemical cycles of the soil system (Steinsham *et al*. 2004). This was supported by Gou *et al*. (2020), at which, though the enormous application of chemical fertilisers benefited countries in providing large amounts of foods, the extended use however has damaged the ecosystem, human health as well as living environments. Moreover, apart generates negative environmental effects, the surplus use of these chemical inputs is costly (Bhargava *et al*. 2017; Chandini *et al*. 2019). Hence, the challenge is to promote more environmental-friendly agricultural approach for sustainable agriculture.

In regard of this sense, a wide number of studies have been and still being conducted, focusing on plant growth promoting rhizobacteria as a potential bioinoculant of fertilisers, herbicides and fungicides (Adesemoye *et al*. 2008). Shaikh *et al*. (2016), in the study also stated a continuous research in searching for eco-friendly management of plant disease along with promotion of plant growth is conducted. Besides, according to Adedeji *et al*. (2020), a strategy without having long term consequences towards the ecosystem, for a sustainable and robust crop production, could be focusing on the utilisation of earth microbe. Such an integrated approach will help in sustaining soil health and productivity and thus there is a need to advocate the biofertiliser approach among the farming community.

As described by Backer *et al*. (2018), microbes assist in plant nutrient acquisition act via various mechanisms included nitrogen fixation, phosphate solubilisation, as well as production of siderophore and hydrogen cyanide (HCN). Thus, manipulating these microbial activities has a great potential in providing crop’s nutritional requirements. Therefore, the major emphasis is being stressed on the exploitation of the biodiversity of plant growth promoting rhizobacteria (PGPR) such as nitrogen fixers and as phosphate solubilisers (Bagyaraj & Aparna 2009).

PGPR which also termed as plant health promoting rhizobacteria (PHPR) or nodule promoting rhizobacteria (NPR) are associated with rhizosphere, an important soil ecological environment for plant-microbe interactions (Hayat *et al*. 2010). Tang *et al*. (2020) also described PGPR possess beneficial traits or cross functional abilities which essential in improving plant’s growth. It capable of promoting the plant growth through multiple mechanisms of actions; either directly, by production of substances which promote plant growth and increase the nutrient availability, or indirectly, by suppressing the plant pathogens in the rhizosphere (Ribeiro & Cardoso 2012; Glick 1995). These PGPR traits, hence, were expected to be a helpful in producing bioinoculant of biofertiliser.

Although the studies related to PGPR are relatively advanced in agricultural systems, research on these bacterial groups in Palmae crops such as sago palm (*M. sagu*, Rottb.) still requires much further study. Hence, the objective of this study is to isolate and identify the native PGPR of sago palm for the contribution of beneficial information for potential bioinoculant of biofertiliser development.

## MATERIALS AND METHODS

### Soil Samples Collection

The soil samples were collected from Dalat and Kuching, Sarawak, Malaysia. Three soil samples were collected from Dalat area, namely Sungai Nunau (N2°45′14.32526″, E111°56′48.61795″), intermediate of Sungai Taap and Sungai Petah (N2°45′11.40469″, E111°56′47.24556″) as well as Sungai Ugui (N2°46′41.9992″, E111°56′1.0108″). Another soil sample was collected from Sago Research Plot Kuching (N1°24′05.9″, E111°20′16.7″). The samples were collected from the depth of 0 cm–15 cm and stored at 4°C–8°C in Bacteriology Laboratory of the Department of Molecular Biology, Universiti Malaysia Sarawak (UNIMAS) before being processed.

### Isolation of Potential Plant Growth Promoting Rhizobacteria

The isolation of potential diazotrophic bacteria was done by using serial dilution spread plate (Deora *et al*. 2006; Islama *et al*. 2007) on Nutrient Agar (Merck Millipore, UK). Approximately 10 g of soil samples were transferred to 250 mL conical flask containing 90 mL of sterile phosphate buffer saline solution (Amresco, USA) and were shaken by orbital shaker (New Brunswick Scientific, USA) at 120 rpm for 30 min. The homogenates samples were diluted up to 10^−3^ dilution on nutrient agar and incubated at 28 ± 2°C for 24 h. The population of bacteria was expressed as colony forming units (CFU) and single colony of each isolates, then were subculture several times on the same agar media to obtain pure PGPR bacterial cultures.

### *In vitro* Screening of Bacterial Isolates for PGP Traits

#### Nitrogen fixation

The nitrogenase activity of the isolates was determined by the growth on nitrogen-free medium, the Burks Agar (HiMedia, India) according to Burk (1930). Pure bacterial colonies were streaked on the Burks Agar and incubated for 5–7 days at 28 ± 2°C. The appearance of the bacterial colonies indicated a positive test.

### Phosphate solubilisation

Screening of phosphate solubilising activity by all isolates was done on Pikovskaya agar, PVK (HiMedia, India), containing 0.5% Ca_3_(PO_4_)_2_ as P source (Pikovskaya 1948). About 30 mL of optimum grown bacteria culture (OD 0.08–1.0 at 600 nm wavelength) was inoculated on double layered sterilised filter paper. The filter paper then was placed on the PVK. The inoculated PVK plates were incubated at 28 ± 2°C for 7 days. Only isolates surrounded by clear halos were considered as phosphate solubilisers. Their phosphate solubilising efficiency and solubilising index was calculated using following formula:


$$\text{Solubilising efficiency }(\text{SE})=\frac{\text{Z}-\text{C}}{\text{C}}\times 100\%$$

where,

Z = Solubilisation zone (cm)
C = Colony diameter (cm)

$$\text{Solubilising index }(\text{SI})=\frac{\text{Solubilisation zone}+\text{Colony diameter}}{\text{Colony diameter}}$$

### Production of Indole Acetic Acid (IAA)

Indole acetic acid production was quantitatively measured according to Gordon and Weber (1951). Bacterial cultures were grown in test tubes, each containing 5 mL nutrient broth (Merck Millipore, UK) amended with 0.1% (w/v) tryptophan, incubated at 28 ± 2°C for 24 h. Nutrient broth was used instead of yeast extract mannitol medium (YEM) as described in Gordon and Weber (1951) method. Then, the cultures medium was centrifuged at 10,000 rpm for 10 min. The total of 1 mL supernatant was mixed with 2 mL of Salkowski reagent. Tubes were incubated in dark at room temperature for 25 min. The development of pink colour indicates high production of IAA and the intensity of pink colour was read at 530 nm wavelength. The concentration of IAA produced, then, was extrapolated from the standard curve (Gordon & Weber 1951).

### Identification of Bacteria Using 16s rRNA Gene Partial Sequences

Isolates DNA was extracted by boil cell method as described by Freschi *et al*. (2005). The 16s rRNA gene amplification was performed by using universal primer 356F (5′ ACWCCTACGGGWGGCWGC) and 1064R (5′ AYCTCACGR CACGAGCTGAC). The PCR amplifications were performed with 50 μL PCR Reaction Master Mix containing 10 μL of 5× Green GoTaq ® Flexi buffer, 6 μL of 25 mM MgCl_2_, 3 μL of 10 mM dNTPs, 8 μL of sterile distilled water, 20 μL sample of 20–40 ng DNA, 1 μL of GoTaq® Flexi DNA polymerase, 1 μL of each forward primer and reverse primer. This PCR amplification then performed by thermal cycler (SensQuest, Germany) at an initial denaturation temperature 95°C for 5 min, 35 cycles of; denaturation at 95°C for 30 s, annealing at 60°C for 30 s and extension at 72°C for 160 s followed by a final extension at 72°C for 5 min (Winsley *et al*. 2012). Then, each reaction was analysed by agarose gel electrophoresis in 1.0% (w/v) agarose gel and ethidium bromide (Promega, USA) staining. The electrophoresis conditions were done at 90 V with 200 mA for 30 min before visualised under UV transilluminator (Maestrogen, TW).

The purification of DNA was performed using QIAquick® Gel Extraction Kit (Qiagen, Germany) following the QIAquick Gel Extraction Kit Protocol (Kathleen et al. 2014). The purified DNA fragment, then were sent to 1st Base Laboratory Sdn. Bhd. Malaysia for sequencing purposes. The sequences obtained, then were subjected to Basic Local Alignment Search Tool (BLAST) analysis and nucleotide sequence similarities were determined with the aid of National Center for Biotechnology Information (NCBI) database.

## RESULTS

A total of 17 isolates were successfully isolated from the rhizosphere soils of *M. sagu*, Rottb. of Dalat and Kuching area (Table 1).

As shown in Table 1, the highest number of potential PGPR isolates was located at Sungai Ugui, Dalat, followed by Sago Research Plot, Kuching, Sungai Nunau, Dalat and Intermediate of Sungai Taap and Sungai Petah (Dalat) with a total number of 7, 5, 3 and 2 isolates, respectively. Results also showed that the sampling sites with wild sago palm history namely Sungai Ugui, Dalat, have higher numbers of isolates from total of isolates (41%) compared to new sago palm cultivation sites at Sago Research Plot, Kuching, Sarawak (29%).

### Morphological Characterisation of Bacterial Isolates

These five isolates were subjected to morphological and biochemical characterisation prior to *in vitro* screening of PGP traits (Table 2).

Based on Table 2, all isolates were Gram-positive, whereby they appeared in purple colour with either bacilli or cocci characteristic. The morphological of the bacterial isolates were also determined, at which all isolates appeared in round shape while exhibited various range of sizes and colony colours. In terms of catalase activity, all isolates were found to react positively for catalase assay.

### *In Vitro* Screening of Bacterial Isolates for PGP Traits

About five isolates of these bacteria isolates showed positive tests for all screening tests of PGP traits (Table 3).

Based on Table 3, a total of 5 isolates (31.3%) possessed multiple beneficial PGP traits; as the nitrogen fixer, phosphate solubiliser and IAA producer.

In terms of nitrogen fixation test, all five isolates showed positive reaction with the presence of growth of each isolate on nitrogen free media, Burks agar.

Meanwhile, the phosphate solubilising efficacy of each isolates was directly proportional to the solubilisation zone on Pikovskaya agar after incubation for 5 to 6 days at 28 ± 2°C. The five isolates were shown to response positively, at which S13 isolates solubilised the highest amount of phosphate, followed by S3A isolate, S42 isolate, S2B isolate and S3C isolate, with phosphate solubilising index value of 4.50, 2.67, 2.50, 2.33 and 2.33, respectively.

On the other hand, in IAA production screening test, all five isolates produced IAA significantly with the highest IAA production by S3C isolates; followed by S2B, S42, S3A and S13, which produced 17.30 μg/mL, 10.39 μg/mL, 1.53 μg/mL, 1.38 μg/mL and 1.04 μg/mL, respectively (Table 3).

### Identification of Bacteria Using 16s rRNA Gene Partial Sequences

As shown in Table 4, BLAST searches against the NCBI nucleotide database revealed the isolates close relationship to known plant-associated bacteria, genera *Bacillus* and *Lysinibacillus*.

## DISCUSSION

PGPR does involve as a factor contributing to growth promotion, crop protection as well as in the improvement of *M. sagu*, Rottb. soil health. As rhizosphere soil live in close association with the roots, it will benefit the growth of *M. sagu*, Rottb, by direct secretion of plant growth hormones and plant stimulators in their vicinity. Thus, the isolation and identification of the potential PGPR from *M. sagu*, Rottb. grown in Dalat and Kuching area was decided.

A total of 17 isolates were successfully isolated from four soil samples. However, out of all isolates, only five isolates were chosen to further the molecular characterisation, based on their ability to meet the PGPR traits. All isolates were found to be Gram-positive, which indicated by the purple stained after Gram staining and upon observation under compound light microscope. Isolate S13 and S3A which were collected from Sungai Nunau and Sungai Ugui appeared to be Gram-positive cocci, while the remaining three isolates were Gram-positive bacilli. The same research concerning the same field of interest was conducted in the Philippines by Labrador *et al*. (2014), reported to obtain mostly Gram-negative bacilli isolates, which differed to the isolates isolated in this study. This is might due to the great diverse of the bacteria divisions.

All isolates were found to react positively with nitrogen fixing screening test, as all isolates were found to be growing well on nitrogen free medium, the Burks Agar. The results obtained, thus, corresponded to the diazotrophic characteristic of PGPR, whereby its capability in fixing atmospheric nitrogen. The Burks Agar was designed in such a way that it is lacking in nitrogen source, but the carbohydrate source as well as inorganic salts was remained. Thus, when being introduced on the medium, these isolates were expected to be capable in utilising the atmospheric nitrogen for their cell protein synthesis purposes, which imitate the actual phenomenon undergone by PGPR. The resulted cell protein then, being mineralised into the soil once the senescence of cells occurs. Hence, it will contribute towards the nitrogen availability of the *M. sagu*, Rottb.

Next to their ability in nitrogen fixing, the isolates have also been found to solubilise phosphate *in vitro*. The isolate S13 was found to solubilise the highest amount of phosphate through the largest halo zone formation (μ ≈ 2.1 cm) on the Pikovskaya Agar. This medium was modified in such a way that the insoluble tricalcium phosphate is present, imitating the natural phenomenon faced by most of the plants. The tricalcium phosphate will be converted into soluble form once phosphate solubilising bacteria introduced into the medium. This resulted in the formation of clear halo zones. This beneficial to *M. sagu*, Rottb. development as the application of phosphate fertiliser could be reduced up to 50% without any significant reduction on the yield. This is so because, the rhizosphere could assimilate the soluble phosphate, which eventually preventing the adsorption and fixation in the soil, thereby, supplying the phosphates to the crops. Therefore, these phosphate-dissolving bacteria play a part in correcting phosphorus deficiency of the crop plants (Subba 1977).

IAA is one of the members of phytohormones group, considered as important auxin in plant growth promotion. Out of all isolates tested for IAA production, only two isolates, S2B and S3C were recorded to give significant higher value in phosphate solubilising index and solubilising efficacy. Earlier studies reported that, most of the IAA producing strains are Gram negative, which is differed to the findings obtained in this study (Lindow *et al*. 1998; Datta & Basu 2000). However, similarly, a study by Wahyudi *et al*. (2011) found that few Gram-positive strains belong to Bacillus strain are known to produce IAA. These are supported by Nacoon *et al*. (2020), whereby *Bacillus* genera has found to capable in producing high amount of IAA. As in this study, the isolate S3C, characterised as Gram-positive bacilli was found to be the most efficient IAA producer.

The ability of isolate S3C to use L-tryptophan supplemented into the medium is one of important factor that influence the isolates in producing IAA. This was supported by Ghosh *et al*. (2015), which found that bacteria preferred L-tryptophan for growth and IAA production. It also found that with an increase production of tryptophan, the production of IAA increases proportionally. Wahyudi *et al*. (2011) in his study also stated that tryptophan is the main precursor in IAA biosynthesis via the indole pyruvic pathway (IPA) pathway.

Upon the amplification of 16S rRNA sequences using 356F and 1064R on S3C isolates, it was identified that it belongs to *Bacillus thuringiensis* with 99% identity value. Previous research by Qi (2016) has similarly reported the same strain, at which *Bacillus thuringiensis* found to be an effective bio insecticide in tomato. In the study, it was found that seed germination and shoot elongation was observed upon treating tomato seeds with the bacterial culture filtrate and bacterial suspension, indicates *B. thuringiensis* as effective PGPR.

On the other hand, S2B was identified as *Lysinibacillus sphaericus* strain LSR1, with 99% identity value. A study on rice-associated *Bacillus*, by Shrestha *et al*. (2016) found that some strains closest to *Lysinibacillus* sp. found to be one of potential bio control agents. These are due to their ability to supress disease development of sheath blight and bacterial panicle blight in rice. However, in the study, it was stated as well that these closest strains of *Lysinibacillus* sp. did not show visible antagonistic activities, which remains to be tested in further research. Vendan *et al*. (2010), on the other hand, found *Lysinibacillus* sp. to be one of the most positive rhizobia in having plant growth promoting traits, indicates their roles in growth promotion of ginseng.

## CONCLUSION

Throughout this study, it could be concluded that the plant growth promotion among the isolates were attributed to their individual competencies. The potential rhizobacteria was screened for their plant growth promoting properties and it turned out that two isolates (S3C, S2B) found to be the most effective isolates and were identified as *Bacillus thuringiensis* and *Lysinibacillus sphaericus*. The current study effort is towards aiding research on potential biofertiliser agent for economically important palm, particularly sago palm, which used as a plantation crop in Sarawak, Malaysia for starch production. Thus, to evaluate the influence brought by the most promising bacterial strains found in this study, a pot experiment on the sago seedlings should be further conducted.

## Figures and Tables

**Table 1:** List of isolates from four rhizospheric soil samples.

Sample no.	Isolate code	Locations	Coordinates	No. of isolates	%
Sample 1	S1, S2, S3	Sungai Nunau (Dalat)	N2°45′14.32526″ E111°56′48.61795″	3	18
Sample 2	SA, SB	Intermediate of Sungai Taap and Sungai Petah (Dalat)	N2°45′11.40469″ E111°56′47.24556″	2	12
Sample 3	S3A, S3C, S1, S2, S3, S4, S5	Sungai Ugui (Dalat)	N2°45′54.62053″ E111°56′15.17129″	7	41
Sample 4	SR1, SR3, SR4, SR5, SR6	Sago Research Plot (Kuching)	N1°24′05.9″ E111°20′16.7″	5	29

Total isolates				17	100

**Table 2:** Morphological characteristics of selected isolates.

Isolates	Size	Shape	Colour	Gram’s reaction	Catalase activity
S13	Medium	Round	Light pink	Gram-positive Cocci	+
S3A	Medium	Round	Light yellow	Gram-positive Cocci	+
S42	Large	Round	White (Solid)	Gram-positive Bacilli	+
S2B	Small	Round	White (Solid)	Gram-positive Bacilli	+
S3C	Small	Round	White (Opaque)	Gram-positive Bacilli	+

**Table 3:** Biochemical analysis and *in vitro* screening of potential PGPR isolates.

Isolates	Biochemical analysis		Screening of PGPR traits		
	
	Gram staining	Catalase activity	Nitrogen fixation	PSI	IAA production (μg/mL)
S13	Gram-positive Cocci	+	+	4.50	1.04
S3A	Gram-positive Cocci	+	+	2.67	1.38
S42	Gram-positive Bacilli	+	+	2.50	1.53
S2B	Gram-positive Bacilli	+	+	2.33	10.39
S3C	Gram-positive Bacilli	+	+	2.33	17.30

*Note*: PSI = Phosphorus solubilisation index

**Table 4:** DNA sequencing result of S2B and S3C isolates.

Isolate code	Species	Identity value (%)	NCBI accession number
S2B	*Lysinibacillus sphaericus*	99	MF000302.1
S3C	*Bacillus thuringiensis*	99	HG799991.1
